# Vitamin D Auto-/Paracrine System Is Involved in Modulation of Glucocorticoid-Induced Changes in Angiogenesis/Bone Remodeling Coupling

**DOI:** 10.1155/2020/8237610

**Published:** 2020-09-04

**Authors:** Olha Lisakovska, Ihor Shymanskyi, Dmytro Labudzynskyi, Anna Mazanova, Mykola Veliky

**Affiliations:** Department of Biochemistry of Vitamins and Coenzymes, Palladin Institute of Biochemistry of the National Academy of Sciences of Ukraine, Kyiv, Ukraine

## Abstract

Osteoporosis is a devastating side effect of chronic glucocorticoid (GC) treatment. Despite the crucial role of vitamin D (VD) in bone homeostasis, the precise molecular mechanisms of its action on GC-induced disturbances of bone remodeling remain undefined. The study was performed to elucidate the relation of VD status to GC-induced changes of the angiogenesis/osteogenesis/bone resorption coupling in bone tissue. Female Wistar rats received prednisolone (5 mg/kg of b.w.) with or without VD_3_ (1000 IU/kg of b.w., for 30 days). Biomechanical parameters of rat femurs were assessed by the three-point bending test. The levels of calcium, inorganic phosphate, activity of total alkaline phosphatase (ALP), and its isoenzymes were determined spectrophotometrically. Vascular endothelial growth factor-A (VEGF-A) and caspase-3 protein levels were detected by western blotting. *Vdr* and *Cyp27b1* mRNAs were measured by qRT-PCR. Receptor activator of nuclear factor *κ*B (RANK) expression in bone sections was visualized immunohistochemically. Serum 25(OH)D was assayed by ELISA. GC administration led to a decrease in maximal load (by 1.2-fold) and stiffness and toughness (by 1.3-fold), which was accompanied by a 3-fold reduction of 25(OH)D level, an elevation of the ALP bone isoenzyme activity in serum, hypocalcaemia, and hypophosphatemia. Along with prednisolone-induced VD deficiency, an impaired synthesis of *Vdr* (−30%) and *Cyp27b1* (+71%) mRNA was observed, reflecting deregulation of bone tissue VD-auto-/paracrine system. GC caused an increase in caspase-3 content, suppressed the synthesis of the osteoclastic marker RANK, and altered angiogenesis/osteogenesis coupling by significantly reducing the level of VEGF-A.VD_3_ treatment restored serum 25(OH)D content and the expression of key components of the VD-auto-/paracrine system. VD_3_ supplementation diminished cell apoptosis and strongly improved angiogenesis/osteogenesis coupling as well as mineral metabolism and biomechanical parameters of femurs in GC-administered rats. Thus, VD_3_ can have a beneficial effect on the correction of GC-induced pathological changes in bone remodeling.

## 1. Introduction

Long-term glucocorticoid (GC) therapy is an effective instrument against a variety of chronic inflammatory diseases. However, secondary osteoporosis is one of the most devastating side effects of prolonged GC administration [1]. The main cause of GC-induced bone loss is an aberrant bone homeostasis-impaired balance between processes of bone formation and bone resorption. Thus, it is an important theoretical and clinical problem to disclose molecular and cellular mechanisms underlying GC-induced osteoporosis and the ways to correct impairments in the bone tissue function induced by chronic glucocorticoid therapy.

Vitamin D is known to play a crucial role in bone remodeling [2]. Mechanism of vitamin D action is complex and consists of several metabolic steps. In the liver, vitamin D is hydroxylated by enzymes CYP2R1 and CYP27A1 at C-25 to produce 25-hydroxyvitamin D (25(OH)D), the major circulating form of vitamin D and the most reliable biomarker of vitamin D status [3]. The next step is the hydroxylation of 25(OH)D at C-1 catalyzed by 25(OH)D-1*α*-hydroxylase (CYP27B1) to form the hormonally active metabolite 1*α*,25(OH)_2_D, which is responsible for the biologic action of vitamin D [4]. Besides the kidneys, it has been reported that CYP27B1 is present extrarenally in a number of tissues [5]. 1*α*,25(OH)_2_D acts via nuclear vitamin D receptor (VDR), thus regulating the expression of more than 500 genes [6]. Local production of 1*α*,25(OH)_2_D and VDR expression in almost all tissues and organs regulate cell growth and differentiation in an autocrine and/or paracrine manner. Vitamin D status is known to be related to bone mineral density and bone turnover [7]. More recently, a significant association has been shown between steroid overload and vitamin D deficiency in a large representative sample of US children and adults [8].

In addition to the proper balance between bone formation and bone resorption, an important role in maintaining bone homeostasis belongs to the process of angiogenesis. Since bone is a rather vascularized organ and angiogenesis is critical for osteogenesis, vascular endothelial growth factor-A (VEGF-A) plays a pivotal role in the development of the skeleton and postnatal bone repair [9]. VEGF-A is responsible for endochondral bone formation and required for effective coupling of angiogenesis and osteogenesis during bone repair [10]. Moreover, it has been recently reported for cancer cells that GCs may repress the expression of proangiogenic factor VEGF [11].

Despite the crucial role of vitamin D and its receptor VDR in bone remodeling, the precise molecular mechanisms of their action on GC-induced disturbances, especially on the angiogenesis/osteogenesis/bone resorption coupling have not yet been fully determined. Thus, the aim of our study was to elucidate the role of vitamin D deficiency/sufficiency in GC-induced changes of the angiogenesis/osteogenesis/bone resorption coupling in rat bone tissue.

## 2. Materials and Methods

### 2.1. Animals

Female Wistar rats (100 ± 5 g) were housed under standard conditions and were allowed free access to standard rodent diet and water *ad libitum*. Animals were acclimated for one week before random allocation to three groups, each including 10 animals: (1) the control group; (2) the group that received synthetic glucocorticoid prednisolone at a dose of 5 mg per kg of body weight (per os, daily for 30 days); (3) the group that received prednisolone at a dose of 5 mg per kg of body weight and 1000 IU of vitamin D_3_ (cholecalciferol) per kg of body weight (per os, daily for 30 days). All experimental procedures were performed in accordance with national and international guidelines and laws concerning animal welfare: European Convention for the protection of vertebrate animals used for experimental and other scientific purposes (Strasbourg, 1986), Bioethical expertise of preclinical and other scientific research conducted on animals (Kyiv, 2006).

### 2.2. Mineral Metabolism Assessment

Serum calcium level was determined using bio-test kit (LAHEMA, Czech Republic). The content of inorganic phosphate was measured after protein precipitation from serum with 12% solution of trichloroacetic acid by the method of Dyce [12]. The activity of total alkaline and acid phosphatase in blood serum was measured using bio-test kits (LAHEMA, Czech Republic). Activity of isoenzymes of alkaline phosphatase (ALP), in particular, the bone thermolabile isoform was determined after incubation of the serum samples at +55°С; L-phenylalanine was used as an inhibitor for the intestinal ALP isoform according to the method described [13].

The content of mineral components in bone tissue was studied after protein extraction from bone tissue by the method of dry mineralization at the temperature of +600°C–800°C. Mineral components in the bone ash were determined spectrophotometrically after dissolving in 0.5 ml of hydrochloric acid and subsequent 20-fold dilution in distilled water.

### 2.3. Three-Point Bending Test

Immediately after the dissection, surrounding tissues (muscles and tendons) were removed and femurs were fixed in Bürkhardt's formaldehyde (12% buffered formaldehyde solution supplemented with methanol and glucose) for two days and after that stored in 70% ethanol. Before the mechanical testing bones were rinsed in PBS for 24 hours. The three-point bending test (span length 5.5 mm, loading speed 0.155 mm/sec) at the rat mid-femur was made by the Instron 3366 universal testing machine (Instron Corp., USA). Based on the recorded load deformation curves, the biomechanical parameters were calculated using Bluehill 2 v2.6 (Instron Corp., USA) and Excel software.

### 2.4. RNA Isolation and Real-Time PCR

Extraction of total RNA from bone tissue was performed using the innuPREP RNA Mini Kit (Analytik Jena AG, Germany). Maxima H Minus First Strand cDNA Synthesis Kit (Thermo Fisher Scientific Inc., USA) was used to synthesize cDNAs samples, which were served as templates for real-time PCR analysis on Standard real-time PCR Thermal Cycler (AnalytikJena AG, Germany). Specific primer sequences for *Vdr*, *Cyp27b1*, and a reference gene glyceraldehyde 3-phosphate dehydrogenase (*Gapdh*) were designed using Primer BLAST software: *Vdr*–forward 5′-TCATCCCTACTGTGTCCCGT-3´; reverse 5′-TGAGTGCTCCTTGGTTCGTG-3´; *Cyp27b1*–forward 5′-TGGGTGCTGGGAACTAACCC-3; reverse 5′-TCGCAGACTGATTCCACCTC-3´; *Gapdh*–forward 5′-TGAACGGGAAGCTCACTGG-3´; reverse 5′-TCCACCACCCTGTTGCTGTA-3´. Target genes were amplified for 60 cycles using Maxima SYBER Green/ROX qPCR Master Mix (Thermo Fisher Scientific Inc., USA). Calculations of relative mRNA expression were performed according to the comparative 2−ΔΔCt method of Livak and Schmittgen [14]. Data were normalized to an internal housekeeping gene *Gapdh* and then calculated as the fold change relative to control.

### 2.5. Western Blot Analysis

The protein levels of VEGF and caspase-3 p17 in bone tissue were determined by western blot analysis. Bone tissue samples were lysed in RIPA buffer containing protease inhibitor cocktail (PIC, Sigma, USA). Lysate samples (50 *µ*g protein) were electrophoresed on 15% SDS-PAGE gel and then transferred onto nitrocellulose membranes. The membranes were blocked and then incubated with anti-VEGF antibody (1 : 500, Santa Cruz Biotechnology, USA) and anti-caspase-3 antibody (1 : 1000, Santa Cruz Biotechnology, USA) overnight at +4°C followed by incubation with secondary antibodies: anti-rabbit IgG (*H* + *L*)-HRP conjugate (1 : 4000; Bio-Rad Laboratories, Inc., USA) and anti-mouse IgG (Fab specific)-Peroxidase (1 : 2500; Sigma, USA) for 1 h at room temperature. Thereafter, the membranes were developed with chemiluminescent agents: p-coumaric acid (Sigma, USA) and luminol (AppliChem GmbH, Germany). VEGF and caspase-3 tissue levels were normalized to *β*-actin (1 : 10000; Sigma, USA). The immunoreactive bands were quantified with Gel-Pro Analyzer v3.1 software.

### 2.6. Immunofluorescence Staining and Confocal Microscopy

The rat femurs were cleared of surrounding tissues and the bone marrow was washed from the femur. Then, the bone was fixed with a 10% formalin solution for 48 h, washed, and fixed with a mixture of ethanol and formalin (1 : 10) followed by ethanol of increasing concentration (60–96%). After fixation, decalcification with 5% EDTA solution (pH 6.5) was performed. After decalcification, samples were washed and incubated in 95% and 100% ethanol (twice for 30 min), in benzene for 30 min, and poured into paraffin at +58°C. Femur sections were obtained on a rotary microtome. For immunohistochemical labeling, samples were deparaffinized sequentially in xylene (3 × 5 min), absolute ethanol (3 × 5 min), 95%, 85%, and 75% ethanol (3 min for each), and washed with distilled water. The epitopes of antigens were unmasked by incubating in citrate buffer (10 mM, pH 6.0) at +95°C for 10 min, washed, and blocked with 5% bovine serum albumin (BSA) in PBS for 1 h. Then, bone sections were incubated with anti-RANK (receptor activator of nuclear factor *κ*B) antibody (1 : 150; Santa Cruz Biotechnology, USA) overnight at +4°C. After washing, the samples were incubated with secondary DyLight 488-conjugated goat anti-rabbit IgG antibody (1: 500; Thermo Fisher Scientific Inc., USA) for 45 min in the dark box followed by washing with PBS. Additionally, Hoechst staining was performed to visualize cell nuclei and the sections were applied to a slide and covered with cover slip. Diode 405-30 laser (for blue excitation dye Hoechst) and Tunable Argon 458/477/488/514 nm at 30 mW laser (for green excitation dye DyLight 488) were used. Fluorescence was detected using the 420–480 nm and 505–530 nm channels, respectively. Images were acquired using Carl Zeizz LSM 510 Meta confocal laser scanning microscope (Carl Zeizz, Germany) at 400x of magnification and processed using Zeiss LSM Image Browser software.

### 2.7. Serum 25(OH)D Measurement

Commercial ELISA kit (The IDS 25-Hydroxy Vitamin D EIA, USA) was used for quantification of 25-hydroxyvitamin D (25(OH)D) level in rat serum. Measurements of 25(OH)D content were performed according to manufacturer's protocol.

### 2.8. Statistics

The results of all experiments are expressed as mean ± SEM for at least seven rats per group. Each experiment was repeated three times. The hypothesis of normality distribution of data was tested by the Shapiro-Wilk test. Statistical differences between the groups were compared using the ANOVA test. Differences were considered to be significant when *p* ≤ 0.05. All statistical analysis was performed using Origin Pro 8.5 software (OriginLab Corporation, Northampton, MA, USA).

## 3. Results and Discussion

Long-term use of GCs is associated with the development of pathologic complications, the most common of which is glucocorticoid-induced osteoporosis (GIO). To confirm disturbances of bone homeostasis in animals after long-term prednisolone administration, we first evaluated changes in mineral metabolism. The results presented in Table 1 show that prednisolone administration led to a decrease in the total level of calcium due to its biologically active fraction (ultrafiltered) and inorganic phosphate in serum by 17% and 14%, respectively, compared with the corresponding parameters of control animals. Prednisolone also caused a slight decrease in the content of calcium and inorganic phosphate in the tibia ash. Glucocorticoid-induced hypocalcaemia and hypophosphatemia were accompanied by an increase in the overall activity of a valid marker of bone formation, an alkaline phosphatase (ALP), which was 1.5 times higher in the prednisolone group than in the control group (Table 1). Prednisolone elevated the activity of bone ALP isoenzyme by 1.7 times that may indicate prednisolone-induced disturbances in the structural and functional state of bone tissue. An increase in ALP activity could be partially attributed to an increased synthesis of this enzyme in bone tissue, which in turn may be associated with an elevated number of chondrocytes, since glucocorticoids can inhibit osteoblast differentiation and cause osteoblast apoptosis [15].

Other than the bone ALP isoform, it was also important to determine the contribution of the intestinal ALP isoenzyme to the total serum ALP activity, since intestinal ALP is secreted into the serum, where it remains biologically active. The intestinal brush border enzyme ALP has been reported to play a key role in controlling calcium absorption, inhibiting lipopolysaccharides, and maintaining normal gut microbiota [16]. Therefore, we considered it necessary to not only investigate the effect of glucocorticoids on the actual state of bone remodeling, but also evaluate the indirect mechanisms of their action on bone loss due to intestinal disturbances and, as a consequence, impaired calcium absorption. Long-term administration of GC resulted in 1.5-fold increase in the activity of intestinal ALP isoenzyme in blood serum, indicative of its enhanced release from enterocytes into the bloodstream, most likely due to GC-induced cell destruction. These data demonstrate that GC can cause disruption of bone homeostasis, both directly, by altering bone formation/resorption processes, and indirectly, by affecting bowel function and mineral absorption. However, further research is needed on the exact molecular mechanisms that explain the role of ALP in facilitating mineral absorption in order to better understand the role of the gut in promoting bone health and the impairments that may be induced by prolonged glucocorticoid action.

As vitamin D plays a crucial role in maintaining bone homeostasis, it was expected that it would have a positive effect on the level of mineral components in both serum and bone tissue. Indeed, vitamin D_3_ partially or completely normalized serum calcium and inorganic phosphate levels, as well as mineral deposits in bone tissue compared to glucocorticoids action (Table 1). It was also found that the activity of alkaline phosphatase in blood serum was partially restored after vitamin D_3_ administration. These data may indicate a significant role of vitamin D in the correction of impaired mineral metabolism and bone homeostasis, preventing increased bone demineralization after chronic administration of prednisolone.

To further confirm the development of secondary medication-induced osteoporosis and to determine whether the disturbances of bone metabolism affect bone structure characteristics, we used the three-point bending test to assess basic biomechanical parameters of rat femurs. Based on the recorded load deformation curves, the following biomechanical parameters were calculated: maximal load at failure (N), stiffness (N/mm), and toughness (mJ). These are the structural mechanical parameters that characterize the state of the bone as “whole,” namely, the mechanical properties of the femur diaphysis with a gradual load increase on it. Maximal load refers to the greatest load (or force) achieved before fracture and is reported in units of Newtons (N). This is the simplest parameter and it depends on both bone morphology and bone material. Whole-bone stiffness characterizes how much the entire bone deforms when loaded and shows the maximum load level (as experienced during physiological loading) that do not damage the material, and therefore the bone returns to its original state upon unloading [17]. According to the load-displacement curve, stiffness is measured in units of load per displacement (N/mm) and the slope of the elastic region represents the extrinsic stiffness, which is closely linked with the mineralization of the bone [18, 19]. Toughness is a measure of the amount of energy needed to cause fracture [20]. It reflects work per unit of material needed to be done before fracture in mJ.

As indicated in Table 2, chronic prednisolone administration induced a decrease in all three parameters: maximal load at failure by 1.2-fold and stiffness and toughness by 1.3-fold. These data correlate with impaired bone metabolism and suggest increased bone fragility caused by glucocorticoid load, probably even at low degree of physical activity.

The femurs of rats that received vitamin D_3_ in parallel with prednisolone had the higher maximal load (by 1.12-fold) and toughness (by 1.42-fold) values and showed no statistically significant difference in stiffness parameter compared with the control. These data indicate a partial normalization of whole-bone biomechanical parameters of femurs of osteoporotic rats under the influence of osteoprotective compound vitamin D_3_, which is in complete agreement with the previous data regarding the partial normalization of bone mineral metabolism after cholecalciferol administration. However, the recovery of biomechanical parameters occurs much slower than normalization of the content of mineral components in bone tissue. This can be explained by the fact that the mechanical behavior of the entire bone reflects the integrated contribution of bone morphology and mechanical properties at the tissue level, and, in addition, the mechanical properties depend on the balance between organic and mineral compounds.

**Figure 1:**
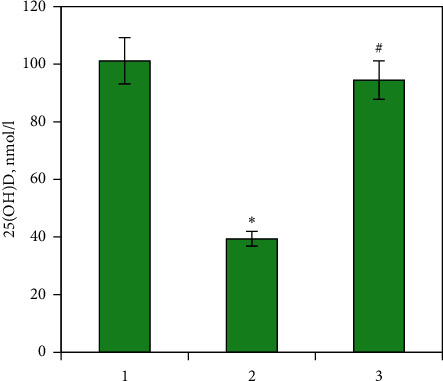
25-Hydroxyvitamin D level in blood serum after prednisolone and vitamin D_3_ administration. 25(OH)D concentration was evaluated by ELISA in rat serum of three animal groups: (1) control; (2) prednisolone administration; (3) prednisolone and vitamin D_3_ administration. All data are presented as mean ± SEM of three independent experiments done in triplicate; ^*∗*^*p* < 0.05 vs. control; ^#^*p* < 0.05 vs. prednisolone administration.

It has been recently reported that the development of GC-induced osteoporosis may be associated with vitamin D deficiency due to impaired vitamin D metabolism in the liver [21]. It can be explained by the fact that vitamin D is an important regulator of bone turnover. One of the main biological functions of vitamin D is to ensure the process of bone remodeling. It regulates mineral metabolism and promotes the deposition of calcium in bone tissue and low vitamin D level may lead to impaired osteoblastic/osteoclastic cell balance. We found that the negative changes in the biomechanical parameters of rat femurs after chronic GC treatment were accompanied by a significant reduction of 25(OH)D (by 2.46-fold) level in blood serum compared with the control, reflecting severe vitamin D deficiency (Figure 1).

Bone tissue is a classic target tissue for the regulatory action of 1*α*,25(OH)_2_D. VDR is expressed in osteoblasts, osteocytes, chondrocytes, and osteoclasts at the early stages of cell differentiation [22]. CYP27B1 is expressed in all types of bone cells except osteoclasts. Thus, the expression of these components of vitamin D auto-/paracrine system can largely reflect the process of osteogenesis (bone formation). Therefore, it was important to evaluate the status of the vitamin D auto-/paracrine system in bone tissue, based on the study of the levels of CYP27B1 and VDR.

**Figure 2:**
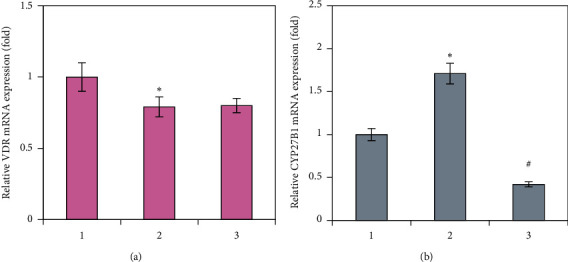
Effects of prednisolone and vitamin D_3_ administration on *Vdr* and *Cyp27b1* mRNA expression in bone tissue. *Vdr* (a) and *Cyp27b1* (b) mRNA levels were determined by quantitative RT-PCR in bone tissue of three animal groups: (1) control; (2) prednisolone administration; (3) prednisolone and vitamin D_3_ administration. mRNA levels were normalized to *Gapdh* expression. All data are presented as mean ± SEM of three independent experiments done in triplicate; ^*∗*^*p* < 0.05 vs. control; ^#^*p* < 0.05 vs. prednisolone administration.

We established that prednisolone administration caused a decrease in *Vdr* mRNA level by 1.37-fold that may contribute to antiosteoblastic effects of GC (Figure 2(a)). This is consistent with the previous results indicating lower VDR protein level in bone tissue after prednisolone load [23]. Reduced VDR level in bone tissue may be attributable to a possible decrease in cell responsiveness to vitamin D action and, as a result, impaired local auto-/paracrine regulation of cell function by vitamin D. At the same time, a 1.71-fold increase in the expression of *Cyp27b1* mRNA in bone tissue was shown (Figure 2(b)), which suggests a compensatory response to lower levels of the components of the vitamin D auto-/paracrine system, 25(OH)D and VDR.

As expected, vitamin D_3_ supplementation increased serum 25(OH)D levels to values in the control group (Figure 1). Surprisingly, no statistically significant changes were detected in the *Vdr* mRNA level after vitamin D_3_ treatment (Figure 2(a)). Meanwhile, vitamin D_3_ reduced the level of *Cyp27b1* mRNA by four times compared with the prednisolone group and 2.36 times compared with the control (Figure 2(b)). Most likely, the observed effect can be adaptive to normalize VDR signaling in response to the restored 25(OH)D level, which serves as a substrate for 25(OH)D-1*α*-hydroxylase.

**Figure 3:**
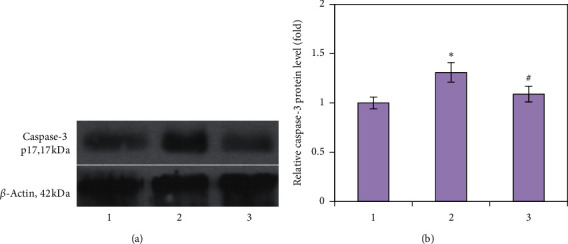
Effects of prednisolone and vitamin D_3_ administration on the protein level of caspase-3 in bone tissue: (1) control; (2) prednisolone administration; (3) prednisolone and vitamin D_3_ administration. Representative immunoblots (a) and quantification of caspase-3 level (b) are presented. Protein levels were normalized to *β*-actin. Results are shown as mean ± SEM of three independent experiments done in triplicate; ^*∗*^*p* < 0.05 vs. control; ^#^*p* < 0.05 vs. prednisolone administration.

Apart from its function in calcium and phosphate homeostasis, vitamin D is known to regulate cell proliferation, differentiation, and apoptosis in numerous tissues [24]. Impaired apoptosis of osteoblasts/osteocytes may lead, at least in part, to the imbalance of bone remodeling resulting in osteoporosis [25]. To confirm the occurrence of apoptotic cell death, we determined the level of one of the most recognized markers caspase-3, a critical enzyme for apoptosis and cell survival. A 1.31-fold increase in the caspase-3 protein level was found, indicative of GC-induced apoptosis activation in bone tissue (Figure 3). However, it is unclear what type of cells undergoes cell death and this is what needs to be clarified in further experiments. Notably, GC-induced elevation of caspase-3 level may contribute to impaired VDR signaling and local auto-/paracrine regulation by vitamin D, since it has been shown that the caspase-3 site is located in a region of the human VDR, and, therefore, VDR may be inactivated by caspase-3 during induction of apoptosis [26].

Vitamin D administration downregulated caspase-3 protein level to the control values, suggesting the possibility of apoptosis regulation in bone tissue cells via direct, through VDR, or indirect mechanisms of vitamin D action. Our data are consistent with previously detected ability of vitamin D to decrease the apoptosis rate in peripheral blood mononuclear cells isolated from patients with systemic lupus erythematosus [27]. It was also reported that administration of vitamin D inhibited intestinal epithelial apoptosis in mice by suppressing the induction of the p53 upregulated modulator of apoptosis (PUMA) apoptotic pathway [28]. Thus, in full agreement with these findings, our data have confirmed that vitamin D can prevent GC-induced apoptosis in bone tissue.

**Figure 4:**
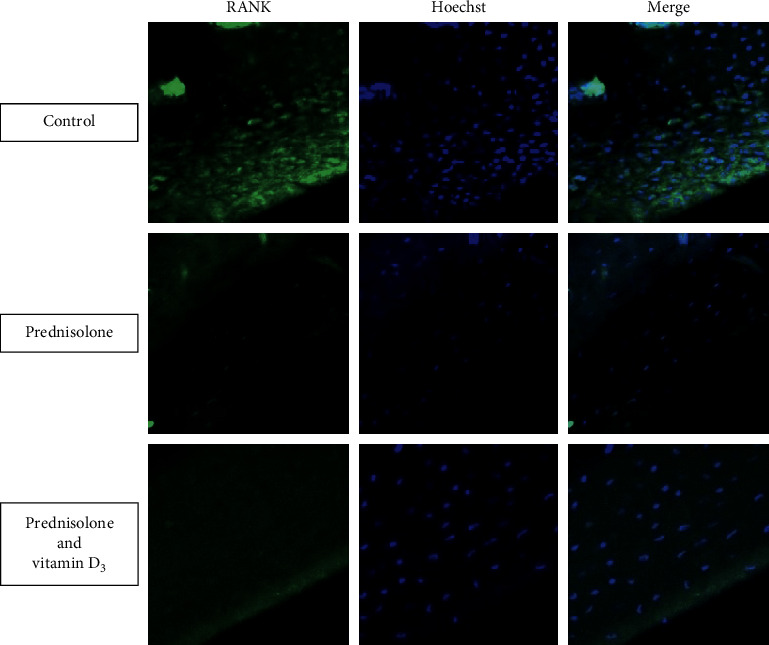
Effects of prednisolone and vitamin D_3_ administration on RANK expression in bone tissue. Immunofluorescence staining of rat femur sections with anti-RANK-antibody (400x magnification) was performed in three animal groups: (1) control; (2) prednisolone administration; (3) prednisolone and vitamin D_3_ administration. All data are obtained in three independent experiments done in triplicate.

To maximize the assessment of changes in bone turnover, we further evaluated the process of bone resorption, which is directly related to bone loss. RANK is one of the well-known bone resorption biomarkers detected on osteoclast precursors and mature osteoclasts in bone tissue [29]. Immunofluorescence staining of rat femur sections with anti-RANK-antibody showed the lowering of RANK level in prednisolone-administered rats (Figure 4). These data are in agreement with our previous results that revealed a decrease in the protein level of RANK in bone tissue [23]. Moreover, the study on BALB/c male mice demonstrated that the number of osteoclasts in the areas of bone destruction was significantly decreased in the GC-treated animals compared with the control group [30].

Next, we estimated the effect of vitamin D supplementation on osteoclasts and found partial normalization of RANK labeling of femur sections. The increased RANK labeling after treatment with vitamin D can be explained by the previously discovered ability of 1*α*, 25 (OH)_2_D to induce RANK mRNA and protein expression in human myelomonocytic cell line, HL60, during the process of differentiation into the macrophage/osteoclast lineage [31]. Additionally, over the past few years, we have demonstrated the effect of vitamin D_3_ coadministered with prednisolone to fully normalize the relative content of RANK protein in the bone marrow cell lysates by diminishing the pool of RANK-positive cells in the bone marrow [32]. We do not exclude the existence of a similar mechanism in bone tissue. Thus, the mechanisms of GC-induced osteoporosis are more complex and intertwined than simply the sum of a decrease in bone formation and an increase in bone resorption.

**Figure 5:**
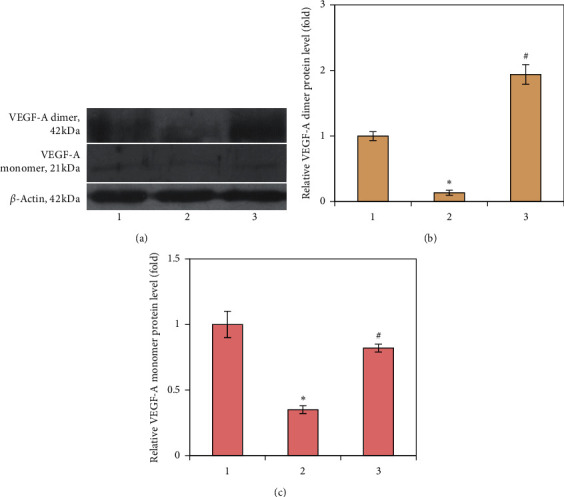
Effects of prednisolone and vitamin D_3_ administration on the protein level of VEGF-A in bone tissue: (1) control; (2) prednisolone administration; (3) prednisolone and vitamin D_3_ administration. Representative immunoblots (a) and quantification of VEGF-A dimer (b) and VEGF-A monomer (c) are shown. Protein levels were normalized to *β*-actin. Results are shown as mean ± SEM of three independent experiments done in triplicate; ^*∗*^*p* < 0.05 vs. control; ^#^*p* < 0.05 vs. prednisolone administration.

Despite the impaired bone remodeling process (osteoclast-dependent bone resorption and osteoblast-mediated bone formation), abnormal angiogenesis can also be associated with the development of glucocorticoid-induced osteoporosis. It is known that VEGF is a key regulator of angiogenesis expressed by bone cells (osteoclasts, osteoblasts, and chondrocytes). Moreover, osteoblast-derived VEGF is important for bone development and postnatal bone homeostasis [33]. Therefore, the question we addressed next concerned the possible involvement of VEGF-A in GC-induced bone loss. Levels of both monomeric and dimeric forms of VEGF-A protein markedly decreased (by 2.86- and 7.35-fold, resp.) after chronic GC administration, suggesting impaired angiogenesis/osteogenesis coupling (Figure 5). We can assume that this is due to interruption of VEGF protein synthesis through the direct influence of GC, since glucocorticoids (100 nM dexamethasone) were previously reported to inhibit the expression of VEGF in growth plate chondrocytes [34].

To date, there is conflicting evidence of cross-talk between vitamin D and VEGF. It has been reported that restoring the bioavailability of vitamin D significantly reduced serum VEGF levels that correlated with lower triglycerides in women with polycystic ovary syndrome (PCOS) [35]. In contrast, 1*α*,25(OH)_2_D may cause an increase in the expression and secretion of VEGF from vascular smooth muscle cells *in vitro*. This can be explained by direct binding of VDR, as a transcription factor, to response elements in the promoter of *Vegf* gene [36]. In accordance with this assumption, we found that vitamin D_3_ supplementation leads to a significant increase in the protein level of VEGF-A. The level of an active form of VEGF-A (dimeric) was elevated by 14.26 times compared with the prednisolone group and by 1.94 times compared with the control (Figure 5(b)). Monomeric VEGF-A increased 2.34 times; however, it did not reach the level of the control group (Figure 5(c)).

As previously reported, VEGF can inhibit the activation of caspase-3 [37]; therefore, we can speculate that an increased level of both forms of VEGF could help reduce caspase-3 p17 and prevent apoptosis in bone tissue. Vitamin D supplementation has essential benefits which counter GC-elicited disturbances in angiogenesis and cell proliferation/differentiation and survival in bone tissue. It should be noted, however, that an obvious limitation of our study is that the level of bone tissue caspase-3 alone is insufficient for a comprehensive assessment of GC-induced cell death, which requires a more detailed study of the intensity and mechanisms of cell apoptosis in our further trials.

**Figure 6:**
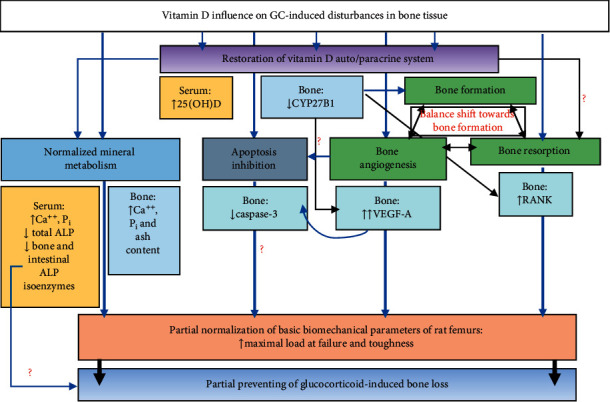
A schematic summary of the results obtained in the study. Osteoporosis was characterized by an impairment of angiogenesis/osteoblastogenesis/osteoclastogenesis coupling that led to a decrease in whole-bone biomechanical parameters of femurs and bone turnover characteristics; vitamin D_3_ demonstrated ameliorative action in prednisolone-induced osteoporosis. 25(OH)D: 25-dihydroxyvitamin D; ALP: alkaline phosphatase; Ca^++^: calcium; CYP27B1: 25(OH)D-1*α*-hydroxylase; *P*_i_: inorganic phosphate; RANK: receptor activator of nuclear factor *κ*B; VDR: vitamin D receptor; VEGF-A: vascular endothelial growth factor A. Blue normal arrows indicate a positive effect; black normal arrows indicate a link/influence;  ⟶: direct;  ↔: bidirectional; ?: points that need to be addressed by future research.

To summarize, the results of our study reveal the link between GC-induced impairments of main processes occurring in bone tissue, which underlie the maintenance of healthy bone homeostasis: angiogenesis by assessing the VEGF level, osteoblastogenesis by determining VDR and CYP27B1, and osteoclastogenesis by RANK labeling. The advantage of the study is the demonstration of the ability of vitamin D_3_ to ameliorate these processes after the deleterious effects of glucocorticoid; thus, restoration of vitamin D bioavailability contributed to the correction of mineral metabolism and biomechanical parameters of rat femurs by diminishing apoptosis in bone tissue, improving osteogenesis/angiogenesis coupling and restoring osteoblastogenesis/osteoclastogenesis balance (Figure 6).

## 4. Conclusions

Our findings showed that prednisolone administration led to a decrease in the level of 25-dihydroxyvitamin D, a vitamin D status marker, in the serum along with an impaired bone synthesis of key components of the vitamin D auto-/paracrine system, VDR and CYP27B1, and consequently induced hypophosphatemia and hypocalcaemia and increased the activity of alkaline phosphatase in the serum and reduced content of mineral components in bone tissue. GC-induced 25(OH)D deficiency and abnormalities in vitamin D metabolism caused a decrease in the synthesis of osteoclastic marker RANK and affected angiogenesis/osteogenesis coupling (reduced VEGF-A levels) that, in general, resulted in osteoporosis and deterioration of the basic biomechanical parameters of rat femurs. Restoration of vitamin D bioavailability contributed to the correction of mineral metabolism and biomechanical parameters of rat bones by diminishing apoptosis in bone tissue, improving osteogenesis/angiogenesis coupling, and restoring osteoblastogenesis/osteoclastogenesis balance (Figure 6). In summary, the present study provides molecular basis for potential usefulness of vitamin D_3_ in the treatment of pathological changes in the process of angiogenesis and bone remodeling associated with the chronic administration of glucocorticoids.

## Figures and Tables

**Table 1:** Bone turnover characteristics of rats with glucocorticoids-induced osteoporosis and after vitamin D_3_ administration, *M* ± *m* (*n* = 10).

Tissue	Characteristic/variable	Control	Prednisolone administration	Prednisolone and vitamin D_3_ administration
Serum	Total calcium, mM·L^−1^	2.23 ± 0.2	1.91 ± 0.05^*∗*^	2.20 ± 0.03^#^
	Protein-bound calcium, mM·L^−1^	0.24 ± 0.03	0.22 ± 0.04	0.21 ± 0.04
	Ultrafiltered calcium, mM·L^−1^	1.99 ± 0.04	1.69 ± 0.03^*∗*^	1.99 ± 0.05^#^
	Inorganic phosphate, mM·L^−1^	2.22 ± 0.06	1.86 ± 0.07^*∗*^	2.26 ± 0.04^#^
	Total activity of alkaline phosphatase, IU/L	233.1 ± 6.3	352.7 ± 9.2^*∗*^	250.5 ± 5.1^#^
	Activity of intestinal isoenzyme, IU/L	44.5 ± 1.68	64.7 ± 1.86^*∗*^	48.3 ± 1.52^#^
	Activity of bone isoenzyme, IU/L	158.2 ± 5.4	264.3 ± 7.2^*∗*^	181.6 ± 5.9^#^

Rat tibia	Ash content, %	56.0 ± 2.4	47.0 ± 1.9^*∗*^	52.0 ± 2.0^#^
	Calcium content, %	30.2 ± 0.29	26.1 ± 0.7^*∗*^	31.6 ± 0.6^#^
	Inorganic phosphate content, %	19.2 ± 0.18	16.3 ± 0.6^*∗*^	18.2 ± 0.5^#^

Values are given as means ± SEM (*n* = 10); ^*∗*^*p* < 0.05 vs. control; ^*#*^*p* < 0.05 vs. prednisolone administration

**Table 2:** Cortical bone strength of rat femurs after prednisolone and vitamin D_3_ administration, *M* ± *m* (*n* = 7).

Groups	Control	Prednisolone administration	Prednisolone and vitamin D_3_ administration
Maximal load at failure (N)	99.9 ± 5.6	80.2 ± 2.3^∗^	89.5 ± 2.8^#^
Stiffness (N/mm)	355.4 ± 10.9	267.2 ± 10.5^∗^	262.8 ± 7.9
Toughness (mJ)	58.3 ± 0.8	43.7 ± 1.2^∗^	62.1 ± 1.4^#^

Maximal load at failure, stiffness, and toughness were analyzed by three-point bending. Values are given as means ± SEM. ^*∗*^*p* < 0.05 vs. control; ^*#*^*p* < 0.05 vs. prednisolone administration.

## Data Availability

The data supporting the findings of the study can be accessed upon a personal request to the corresponding author.

## Supplementary Materials

Supplementary Materials Supplementary table includes the methods used in the study and parameters investigated.
