# Supplementary material for: Vitamin D Auto-/Paracrine System Is Involved in Modulation of Glucocorticoid-Induced Changes in Angiogenesis/Bone Remodeling Coupling
Source: Int J Endocrinol. 2020 Sep 4;2020:8237610. doi: 10.1155/2020/8237610 (PMC7487101; doi:10.1155/2020/8237610)
Supplement: Supplementary Materials — Supplementary table includes the methods used in the study and parameters investigated. [file 8237610.f1.docx]

Table: Methods used in the study and parameters investigated.

| **№** | **Method** | **Sample** | **Parameters** |
| --- | --- | --- | --- |
| 1 | Spectrophotometrical assay | blood serum | - Activity of alkaline phosphatase (ALP): total, bone and intestinal isoforms - Calcium content: total, protein-bound, ultrafiltered - Inorganic phosphate content |
|  |  | bone tissue (femurs and tibia) | - Ash content - Calcium content - Inorganic phosphate content |
| 2 | Three-point bending test | femurs | - Biomechanical parameters:  maximal load at failure, stiffness, toughness |
| 3 | Western blot analysis | Protein lysates from femurs | - Vascular endothelial growth factor (VEGF) - Caspase-3 |
| 4 | Quantitative RT-PCR | RNA from bone tissue | - Vitamin D receptor (*Vdr*) - 25OHD-1α-hydrohylase *(Cyp27b1)* |
| 5 | ELISA | blood serum | - 25-hydroxyvitamin D (25ОНD) |
| 6 | Immunohistochemistry and confocal microscopy | femur slices | - Receptor activator of nuclear factor kappa B (RANK)-positive cells |
